# Rationale and Design of an Ecological Momentary Assessment Study Examining Predictors of Binge Eating Among Sexual Minority and Heterosexual Young Women: Protocol for the Health and Experiences in Real Life (HER Life) Study

**DOI:** 10.2196/41199

**Published:** 2022-10-21

**Authors:** Kristin E Heron, Abby L Braitman, Charlotte A Dawson, Cassidy M Sandoval, Lauren V Butler, Alicia Moulder, Robin J Lewis

**Affiliations:** 1 Department of Psychology Old Dominion University Norfolk, VA United States; 2 Virginia Consortium Program in Clinical Psycholology Norfolk, VA United States

**Keywords:** sexual minority women, ecological momentary assessment, binge eating, sexual minority stress, negative affective states, mobile phone

## Abstract

**Background:**

Previous research has identified health disparities between sexual minority and heterosexual women, including increased rates of obesity and binge eating in sexual minority women. Established predictors of binge eating behavior include negative emotions and sociocultural processes; however, these studies are generally conducted in samples of young women where sexual identity is not known or reported. There is a dearth of research evaluating how sexual minority–specific factors (eg, minority stress and connectedness to the lesbian, gay, bisexual, transgender, and queer community) may affect binge eating in sexual minority women. In addition, no studies have examined these processes in racially diverse samples or considered how intersecting minority identities (eg, Black and sexual minority) may affect eating behaviors.

**Objective:**

The Health and Experiences in Real Life (HER Life) Project aims to clarify real-world predictors of binge eating in young heterosexual and sexual minority women using ecological momentary assessment. The role of affective, social, and health behavior factors in binge eating will be examined for all women (aim 1), and sexual minority–specific predictors will also be considered for sexual minority women participants (aim 2). Person-level moderators of race, body- and eating-related factors, and sexual minority–specific factors will also be examined to better understand how real-world binge eating predictors may differ for various demographic groups (aim 3).

**Methods:**

Researchers aim to recruit 150 sexual minority and 150 heterosexual women from across the United States, including at least 50 Black women for each group, using web-based recruitment methods. The eligibility criteria include identifying as a woman, being aged between 18 and 30 years, and having had at least two binge eating episodes in the last 2 weeks. Participants must endorse being only or mostly attracted to men (considered heterosexual) or only or mostly attracted to women or having a current or most recent female partner (considered sexual minority). Eligible participants complete an initial web-based baseline survey and then 14 days of ecological momentary assessment involving the completion of a morning and before-bed survey and 5 prompted surveys per day as well as a user-initiated survey after binge eating episodes. The data will be analyzed using a series of multilevel models.

**Results:**

Data collection started in February 2021. We have currently enrolled 129 sexual minority women and 146 heterosexual women. Data collection is expected to conclude in fall 2022.

**Conclusions:**

The Health and Experiences in Real Life Project aims to elucidate potential differences between sexual minority and heterosexual women in within-person factors predicting binge eating and inform eating disorder interventions for sexual minority women. The challenges in recruiting sexual minority women, including the determination of eligibility criteria and considerations for remote data collection, are discussed.

**International Registered Report Identifier (IRRID):**

DERR1-10.2196/41199

## Introduction

### Background

Obesity and obesity-related conditions are the leading causes of death in the United States [1], making them a significant public health concern. On the basis of several recent national reports [2,3], there are disparities in the health and well-being of sexual minority individuals (eg, gay, lesbian, and bisexual) as compared with their heterosexual peers in a range of health conditions and health behaviors. In particular, large-scale studies suggest that sexual minority women are at least twice as likely to be obese as heterosexual women [4,5], and studies of young women have similarly found higher rates of being overweight or obese in young lesbian women (35.2%) than in young heterosexual women (22.8%) [6]. There is also evidence that rates of binge eating, defined as overeating with loss of control of eating, are similarly higher in sexual minority individuals. For example, in a study of nearly 14,000 people, young adult lesbian women were twice as likely to binge eat as heterosexual women [7]. Despite these documented disparities, relatively little is known about the contributing factors, which is a critical first step in developing interventions to address these health concerns among sexual minority women.

In working toward increased understanding of factors that contribute to higher obesity and binge eating rates in sexual minority women, the goal of this study is to use ecological momentary assessment (EMA) to identify general and minority-specific real-world predictors of binge eating. EMA involves the use of repeated assessments of people’s current behaviors, states, and experiences as they are going about their lives [8]. EMA approaches provide unique advantages over other research methods (eg, cross-sectional and traditional longitudinal methods), including greater real-world generalizability and reduced concerns regarding memory biases associated with retrospective recall that are generally present in traditional measures. In addition, as EMA uses repeated assessments (typically multiple times throughout the day), researchers can examine dynamic within-person processes and answer research questions that cannot be addressed using other methods [8,9]. For example, most previous research on binge eating among sexual minority women has used cross-sectional designs, which can address questions regarding between-person processes (eg, do women who are more depressed also binge eat more?). EMA approaches provide an opportunity to answer questions regarding *within-person* processes (eg, when women experience a more depressed mood, are they more likely to binge eat?). This study—called the Health and Experiences in Real Life (HER Life) Project—will examine within-person associations between a range of general predictors of binge eating (ie, affect, body dissatisfaction, social processes, and health behaviors) in young heterosexual and sexual minority women as well as sexual minority–specific factors (eg, minority stress and discrimination) among young sexual minority women. The existing empirical evidence and theoretical framework supporting this study are described in the following sections.

### General Factors Associated With Binge Eating in Daily Life (Aim 1)

The factors influencing disordered eating are complex, and researchers have highlighted the potential utility of EMA to study disordered eating, including binge eating, in natural settings [10]. EMA research shows that disordered eating, including binge eating, is associated with affective states (eg, negative mood, stress, and body dissatisfaction) [11,12], social experiences (eg, interactions and media use), and health behaviors (eg, alcohol use and physical activity) in studies of young women where sexual orientation was not assessed or reported (hereafter referred to as general samples). However, with few exceptions [13], EMA studies have yet to consider how affective states, social processes, and health behaviors in daily life are associated with sexual minority women’s binge eating, which is a primary aim of this study.

The role of negative emotions (eg, negative affect and stress) in binge eating is often examined as a way of evaluating the affect regulation model of eating disorders [14]. A meta-analysis of EMA studies evaluating the affect regulation model of binge eating found that negative emotions increased leading up to a binge eating episode, with more mixed findings regarding negative affect after binge eating episodes [12]. Negative feelings about one’s body, or body dissatisfaction, have also been examined via EMA in general samples of young women but, with few exceptions [15], studies have generally focused on associations between body dissatisfaction and social processes, not disordered eating behaviors [16-18]. Although these studies show that negative mood and body dissatisfaction may be associated with binge eating in daily life, this work is largely based on general samples of young women, and whether and how these processes operate in sexual minority women is less clear. There is some preliminary evidence demonstrating that negative affect is associated with binge eating at the day level in a daily diary study of 30 lesbian women [13], but there are no EMA studies of body dissatisfaction in sexual minority women. Cross-sectional studies designed to answer between-person research questions have produced mixed findings, with some suggesting that body dissatisfaction is similar in sexual minority and heterosexual women [19] and others finding that larger body sizes are more acceptable among sexual minority social groups and, thus, body dissatisfaction may be lower [5]. However, as described previously, this study will fill gaps in the literature by assessing negative affect, stress, and body dissatisfaction in daily life to examine how they are associated with binge eating among both young sexual minority and heterosexual women.

There is a long history of research demonstrating the contribution of sociocultural processes to disordered eating behaviors [20,21]. In fact, the sociocultural model of disordered eating suggests that there are social pressures to be thin (and, increasingly, to be fit or toned) in Western countries, which are communicated directly and indirectly through the media, peers, and family and can become internalized (ie, thin ideal internalization) [22]. In recent years, several EMA studies have examined how social experiences are associated with body dissatisfaction and eating behaviors in daily life. For example, in a general sample of 121 women aged 18 to 40 years, women reported greater body dissatisfaction in social situations (ie, when they had had an interaction in the previous 30 minutes) than when alone and particularly when they perceived that the interaction quality was lower [18]. To our knowledge, studies on the effect of social processes in daily life on eating behaviors have been conducted only among general samples of young women and, thus, it remains unclear whether sexual minority women experience the same sociocultural influences as heterosexual women in daily life. Cross-sectional research suggests that larger body sizes may be more acceptable in sexual minority women’s social groups and relationships [5,23] and, thus, they may be less susceptible to thin ideals that they are exposed to in social interactions and the media. However, as described by the dual identity framework, there may also be commonalities between sexual minority and heterosexual women [24]. According to this framework, sexual minority women are influenced, as women, by the mainstream (heterosexual) community and, as sexual minority individuals, by the sexual minority community. Thus, sexual minority women’s eating behaviors will likely be influenced by general social processes but potentially to a lesser extent than heterosexual women. The HER Life Project will explore social influences on both sexual minority and heterosexual women in daily life, thus allowing for a better understanding of how these dual influences may operate for young sexual minority women.

In addition to the demonstrated importance of sociocultural considerations for understanding disordered eating, cross-sectional and meta-analytic studies have shown that hazardous alcohol use is associated with disordered eating in general samples of young adults [25] and among lesbian women. For instance, a cross-sectional survey found that lesbian women with obesity were more likely to report heavy drinking compared with lesbian women who were not overweight or obese [26]. Research on the associations between binge eating and physical activity are more equivocal [27], but 1 study with a general sample of young women found that those with binge eating disorder reported less physical activity compared with weight-matched women who did not have binge eating disorder [28]. Even less is known about physical activity and binge eating in sexual minority women, but 1 study of lesbian and bisexual women found that being overweight or obese was associated with less frequent exercise [29]. It is clear that there is a dearth of research on the associations among alcohol use, physical activity, and binge eating, especially in sexual minority women. This is particularly notable because of the documented disparities in alcohol use and physical activity, with sexual minority women engaging in more hazardous drinking [30-32] and less physical activity [33,34] than heterosexual women. This study will assess alcohol use and physical activity in daily life, which will allow for a real-world examination of how these health behaviors may operate among sexual minority and heterosexual young women, and explore potential associations with binge eating.

### Sexual Minority–Specific Factors Associated With Binge Eating in Daily Life (Aim 2)

In addition to the aforementioned general factors that may influence binge eating, sexual minority women face unique experiences and stressors because of their marginalized and stigmatized status in society. This phenomenon has been described in minority stress theories by Meyer [35] and Hatzenbuehler [36]. These theories complement each other in describing how general and minority-specific stressors can affect mental health and well-being. For example, the minority stress theory by Meyer argues that sexual minority individuals experience stressors related to their minority status. These stressors include chronic and acute external events (discrimination, harassment, invalidations, and vicarious experiences through other sexual minority individuals) and internal stressors (concealment of sexual identity, expected rejection, and internalization of negative societal messages about sexual minorities [internalized heterosexism]) that can negatively affect mental health. The psychological mediation framework by Hatzenbuehler extended these ideas to suggest that general (eg, coping, emotion regulation, and social support) and minority-specific (eg, distal and proximal minority stressors) experiences operate together to influence sexual minority individuals’ well-being. These theories have been extended from their initial focus on understanding mental health to also consider the impact of minority stressors on physical health and health behaviors.

In recent years, minority stress theories have been used to identify potential mechanisms to explain health disparities between sexual minority and heterosexual individuals, including disordered eating. Cross-sectional studies of sexual minority women have found between-person associations between heterosexist experiences (eg, harassment, rejection, and sexual orientation discrimination) and disordered eating [37-39]. More recently, there have been several daily diary and EMA studies that have assessed within-person associations between minority stressors and disordered eating behaviors at the day or moment level. For example, in a sample of 30 young lesbian women who reported binge eating, researchers found that, on days when the women reported more discrimination, they also reported more binge eating (and this effect occurred in part through negative affect) [13]. In an EMA study of 55 sexual minority women who were overweight or obese, Panza et al [40] similarly found that those with higher baseline levels of internalized homophobia and sexual orientation concealment reported more binge eating and overeating during a 5-day EMA period. Taken together, sexual minority stress theories and empirical research suggest that sexual minority stress is associated with poor mental health, with substantially fewer studies considering the effects on binge eating. The goal of this study is to examine the within-person direct and indirect (through negative affect) effects of sexual minority stressors on binge eating in a subsample of sexual minority women. In other words, this study allows us to consider whether, at times when sexual minority women experience a minority stressor, that experience is either concurrently or subsequently associated with binge eating in daily life. Such information may improve the understanding of the potential mechanisms whereby sexual minority women experience more disordered eating behaviors as compared with heterosexual women.

### Risk and Protective Factors for Binge Eating (Aim 3)

Given that theories on binge eating and sexual minority health and health disparities are complex, with factors influencing these behaviors at various *levels*, an additional aim of this study is to consider how *between-person* factors may strengthen or weaken the aforementioned *within-person* associations. Three broad groups of moderators will be considered in this study: race, body- and eating-related factors, and sexual minority–specific factors. Considering these cross-level moderators can be useful for clarifying previous conflicting findings and provide a more complete picture of both *when* and *for whom* various real-world binge eating predictors operate.

Although cross-sectional research shows that sexual minority women are more likely to report binge eating compared with heterosexual women [7,41,42], many of these studies were conducted in samples that were not racially diverse. There is some conflicting research regarding potential racial differences in binge eating between Black and White women in the United States. Some studies in general samples of young women have found no differences in disordered eating (including binge eating) between Black and White women [43,44] but, in a general sample of women with binge eating disorder, Black women reported 1.5 times as many binge eating episodes per week compared with White women [45]. However, taken together, these studies fail to consider how multiple minority identities (ie, being a Black sexual minority woman) might interact to exacerbate health behavior risks, and there is scant research examining the effect of the intersection of sexual and racial identities on binge eating. In fact, we found only 1 study that assessed binge eating in a small, racially diverse sample of lesbian and bisexual women. This cross-sectional study found that 1.6% of White heterosexual women (n=63) reported binge eating at least twice per week compared with rates of 4.5% for White lesbian and bisexual women (n=67) and 3.1% for Black lesbian and bisexual women (n=64) [46]. Importantly, this study was cross-sectional and had a small sample. Therefore, to extend these past findings, in the HER Life Project, we powered to test for Black-White race moderator effects of the within-person associations between the general and minority-specific predictors of binge eating outlined in the first 2 study aims, allowing us to examine how intersecting minority statuses (ie, being Black and of a sexual minority) may uniquely shape eating behavior in daily life.

In addition to considering how women’s racial identity may moderate within-person associations, this study will consider 4 body- and eating-related variables: BMI, person-level (baseline) body dissatisfaction, disordered eating, and thin norm internalization. These variables were selected based on past research demonstrating that individuals higher or lower in these constructs may exhibit different associations between our predictors of interest and binge eating [17].

In addition, this study considers sexual minority–specific moderators of EMA associations, which will be considered among the subsample of sexual minority women. In particular, we will evaluate 3 protective factors (positive sexual minority identity; lesbian, gay, bisexual, transgender, and queer [LGBTQ+] community connection; and identification with the LGBTQ+ community) and one risk factor (lifetime sexual minority discrimination). Previous cross-sectional research suggests that connection to and identification with the sexual minority community may buffer against the deleterious effects of sexual minority stress [47], including perceived stigma and depressive symptoms [48]. Furthermore, in a cross-sectional study of lesbian, gay, and bisexual young adults, social support (eg, from the sexual minority community) attenuated the associations between minority stress and emotional distress [49]. Conversely, lifetime discrimination may be a risk factor for maladaptive health behaviors. In a daily diary study of Black college students, perceived lifetime racial discrimination moderated the associations between negative mood and alcohol use [50], providing evidence that (person-level) lifetime racial discrimination influences daily health behaviors. Taken together, the third aim of this study is to consider a variety of person-level moderators of the within-person associations explored in the first 2 study aims.

### Overview of Study Aims

The broad objective of the HER Life Project is to enhance the understanding of the real-world predictors of binge eating among young sexual minority and heterosexual women. To address this objective, this study is organized around 3 aims.

#### Aim 1: To Examine the Role of Affective, Social, and Health Behavior Factors in Binge Eating

We hypothesize that, when sexual minority and heterosexual women experience higher levels of negative emotion variables (negative affect, general stress, and body dissatisfaction), they engage in more binge eating (hypothesis 1a). We also expect that social experiences (eg, social interactions and media use) will be associated with more binge eating for both groups (hypothesis 1b), but the associations will be stronger for heterosexual women than for sexual minority women (hypothesis 1c). A priori hypotheses for binge eating, alcohol use, and physical activity associations are not made because of limited previous EMA research.

#### Aim 2: To Examine the Associations Between Sexual Minority Stress and Binge Eating Among Sexual Minority Women

Direct within-person associations are hypothesized such that, when sexual minority women experience more sexual minority stress, they will engage in more binge eating (hypothesis 2a). Consistent with theory, we also expect that negative affect will mediate the association between sexual minority stress and binge eating (hypothesis 2b). These aims will be tested within the subsample of sexual minority women recruited for this study.

#### Aim 3: To Explore Race and Other Person-Level Moderators of Associations in Aims 1 and 2

Race, body- and eating-related factors (BMI, body dissatisfaction, disordered eating, and thin norm internalization), and sexual minority–specific factors (lifetime discrimination, positive sexual minority identity, and community identification and connection) will be explored as moderators of EMA associations in aims 1 and 2. Given the scant previous research, no a priori hypotheses are specified, but the study is powered to test for Black-White group differences for aims 1 and 2 to explore the intersection of multiple minority identities.

## Methods

### Project Overview

The HER Life Project is an ongoing EMA study of young sexual minority and heterosexual women who binge eat. Young women aged 18 to 30 years are the focus of this study given that they report high levels of binge eating [4,5] and, thus, represent an at-risk group. We selected women aged 18 to 30 years as, although “young adulthood” has no clear definition, there is evidence that sexual identity continues to develop during this period, and data suggest associations among sexual identity, negative affect, and disordered eating up to the age of approximately 30 years [51]. Participants are being recruited from across the United States, and data collection is occurring entirely remotely as we are using web-based surveys and a smartphone app to deploy EMA surveys. Participants receive and review the written and video study procedure descriptions before beginning the study. They complete a web-based screening and baseline survey before beginning the 2-week EMA data collection period and conclude their participation with an end-of-study survey. During the EMA period, participants complete daily morning and before-bed surveys, 5 prompted surveys each day, and a user-initiated binge eating survey after binge eating episodes.

### Ethics Approval

The Old Dominion University Institutional Review Board approved all the study procedures (project 1362990).

### Power Analysis

A 3-step power analysis was conducted to determine sample size. First, a regression power analysis using G*Power (version 3.1.9) [52] was conducted, powering the hardest-to-detect effects (aim 1 hypothesis 1c: sexual identity moderation; aim 3: race moderation) and the examinations using only sample subsets (aim 2: direct effects, sexual minority women only). To achieve a power of 0.80 using an α of .05, 227 independent observations are required for a small to medium effect (*f*^2^=0.035) for moderation examinations with 5 predictors (to allow for relevant covariates). Specifying the same power level, α, and effect size with 3 predictors for aim 2 main effects (to allow for relevant covariates), 227 independent observations among sexual minority women only would be necessary. Second, we applied a formula to account for the multiple correlated observations for each person and identify the number of actual participants needed [53]. This equation accounts for level-1 (assessments or moments) and level-2 (participants) sample sizes and the intraclass correlation coefficient (ICC; within-person relatedness). On the basis of past EMA studies of similar constructs among young women [54,55], a conservative estimate of the average number of assessments per person (n_L1_) is 25, reflecting constructs only asked once or twice per day (as opposed to all prompts), approximately 70% compliance, and the reporting of approximately 6 binge eating episodes. If 100 sexual minority and 100 heterosexual women are recruited, this would result in approximately 5000 total observations (2500 from sexual minority women). We expect ICC values from 0.33 to 0.43 for binge eating. Using the West et al [53] formula and past studies, n_L2_ of 100 sexual minority and 100 heterosexual women would result in an N-effective of 280 within sexual identity if the ICC is 0.33 and an N-effective of 221 within sexual identity if the ICC is 0.43. This means that, although there are 2500 assessments from sexual minority women, because of the degree of relatedness of observations within each individual, these are equivalent to 221 independent pieces of information. Thus, recruiting 200 women should provide sufficient power for these aims. In addition, to detect small to medium mediation effects (aim 2) using empirical bias-corrected bootstrap CIs, 148 independent observations should be sufficient [56]. Finally, to power for aim 3, half of the 100 participants in each sexual identity group (n=50) will need to identify as Black to optimize power. However, given the demographic representation in previous work examining eating behaviors in young women using EMA [54,55], it is expected that we will need to enroll >100 women of each identity to have 50 Black women in each group even if deliberately oversampling. To ensure that we are sufficiently powered to test all aims and have similar numbers in both groups, we aim to enroll 150 sexual minority women (50 Black) and 150 heterosexual women (50 Black).

### Participant Selection

Participants are being recruited using various web-based sources, including from web-based research panels, social media, and lists of people who participated in past studies and expressed an interest in future research opportunities. We are working with several market research firms who have access to web-based panels of people who are interested in participating in web-based research. Community Marketing & Insights is an LGBTQ+ market research firm that manages a proprietary panel of individuals who identify within the LGBTQ+ community and are interested in participating in web-based studies. Marketing Systems Group and Qualtrics International Inc are also assisting with accessing existing panels of individuals who regularly participate in web-based studies, primarily to recruit heterosexual women. We have also placed advertisements on social media (eg, Facebook and Instagram) using the Meta advertising platform and through advertisements on our laboratory social media page.

To be eligible to take part in the study, participants must (1) identify as women (from a multi-select question, selecting woman or woman *and* some combination of genderqueer, nonconforming, or nonbinary and also indicating that they were assigned female at birth), (2) be aged between 18 and 30 years (inclusive), (3) report binge eating (ie, overeating with a feeling of loss of control of eating) at least two times in the past 2 weeks, (4) not be currently receiving treatment for an eating disorder, and (5) have a schedule that allows for answering surveys during daytime hours. In addition, the participants must also meet either the sexual minority or heterosexual criteria. Women are included in the sexual minority sample if they report that they are (1) only or mostly attracted to women or (2) equally attracted to men and women or attracted to people regardless of their gender identity *and* also have a current or most recent romantic partner who identifies as a woman. Women are included in the heterosexual sample if they report that they are only or mostly attracted to men.

### Study Procedures

#### Recruitment

For participants being recruited through the marketing research firms, the firm makes initial contact with potential participants and administers an initial web-based screening survey. Contact information for the eligible participants is then provided to the research team. At this time, participants are contacted via email and screened directly by the research team using a brief web-based screening survey. For participants being recruited through social media and lists of past research participants interested in research studies, potential participants are provided directly with a web-based screening survey and screened by our team.

#### Informed Consent Process and Baseline Survey

Eligible participants are provided with additional information about the study during the informed consent process. First, a 3-minute video providing a general overview of the study’s purpose, procedures, and participation requirements (eg, downloading a smartphone app) and compensation is emailed to potentially eligible participants. Second, participants are provided with information about how to download the smartphone app needed to complete the EMA surveys. Participants confirm their interest in participating by downloading the smartphone app and notifying the research team that they have done so. The LifeData (LifeData, LLC) survey software and RealLife Exp (LifeData, LLC) app are being used for this study. This software allows for custom EMA surveys and alarm schedules to be created, and the surveys can be deployed on any Android or Apple smartphone using the RealLife Exp app. Plans are in place to provide a phone to participants if they do not have an Android or Apple phone that they are willing or able to use for the study (none have requested this option to date). Participants who do not respond to these emails are sent up to 3 reminders to review the video and download the smartphone app. Additional details regarding the email reminder system used throughout the study process are provided in the following sections. Once participants confirm their interest in proceeding with the study (as evidenced by downloading the app to their phone), they are sent a web-based link to complete an electronic informed consent form, which is immediately followed by the baseline survey, which takes approximately 45 minutes to complete.

#### EMA Surveys

After completing the baseline survey, participants receive an email from our team with instructions on how to download the study-specific EMA smartphone surveys (within the app they previously downloaded) and a document with frequently asked questions and answers about study procedures. Once participants download the study-specific surveys, they complete an initial “startup” session where they enter their study-specific identification number and watch 2 additional brief training videos created by the research team. The first video provides additional information about when and how to complete each type of EMA survey and is approximately 3 minutes long. The second video provides a definition and examples of a binge eating episode to help participants in identifying their binge eating and improve understanding of when to initiate a binge eating survey; this video is approximately 2 minutes long. These informational videos and the document with frequently asked questions and answers are accessible to participants within the smartphone app throughout the entirety of the EMA portion of the study.

The day after the study-specific EMA surveys are downloaded, participants begin to receive 5 prompted surveys per day for the next 14 days at semirandom times between 9 AM and 9 PM each day. In addition to the prompted surveys, participants complete 3 different user-initiated surveys: an after binge eating survey, a morning survey, and a before-bed survey. They are instructed to complete the post–binge eating survey after a binge eating episode; as described previously, participants receive written and video instructions defining, describing, and providing examples of a binge eating episode. They are instructed to self-initiate the morning survey immediately after waking up and the before-bed survey just before going to sleep at night, but the app also provides a reminder notification for each (at 10 AM for the morning survey and 9 PM for the before-bed survey).

#### End-of-Study Survey

After day 14 of the EMA surveys, participants receive an email including a link to complete a survey regarding their experience as research participants in this study. Additional information on this measure can be found in the Measures section.

#### Compensation

Participants can earn up to US $150 for taking part in this study. They receive US $20 for completing the baseline survey and US $10 for completing the end-of-study survey. Each week of the EMA, they receive US $40 (a total of US $80). To incentivize compliance, they can also receive a US $20 bonus each week if they complete 80% of the daily surveys (averaging a morning survey, 4 prompted surveys, and a before-bed survey per day). Within a week of completing the end-of-study survey, participants receive an email thanking them for taking part and notifying them of their compensation amount. Participants receive an electronic gift card (selected from several options) via email at the conclusion of their participation.

#### Email Reminder and Check-in Systems

Given that this study is being conducted entirely remotely and participants need to move through various steps to enroll in and complete the study, we developed a system for reminding participants who do not respond to initial emails or complete tasks as expected. Throughout each stage of the process—screening, completing the baseline survey, beginning the EMA surveys, and completing the end-of-study survey—project staff send participants up to 2 reminders, approximately 2 to 3 days apart, to encourage participation. If participants do not respond to these reminders, a final email is sent by the study principal investigator (PI; KH) to inquire about questions and encourage their continued participation.

In addition to monitoring the overall flow throughout the study, we carefully track compliance with the EMA surveys. Participants’ completion of the daily surveys is tracked and recorded each day using a daily tracking spreadsheet. Participants are sent a general check-in email on day 2 or 3, day 7 or 8, and day 13 or 14 of the EMA portion of the study to thank participants for their completion thus far, offer the opportunity to ask questions, and encourage continued compliance. Participants demonstrating a pattern of noncompliance (eg, no prompted surveys completed in a day, no morning or before-bed surveys completed in a day, or many prompted surveys not completed) receive a noncompliance email. Noncompliance emails are sent to participants to advise them as to which surveys they are not completing on a regular basis, check whether they are experiencing difficulty completing the daily surveys, and encourage participation.

### Measures

The measures in the baseline survey are described in Table 1 and are grouped together by construct. Participants generally complete the same baseline survey, with 2 exceptions. First, there are several specific questionnaires for sexual minority women regarding minority stressors (eg, discrimination and rejection), identity, and LGBT community connection. To balance survey length and content, heterosexual women complete questionnaires regarding their identity as women and their general social support. Second, as we are interested in assessing alcohol use but did not require participants to drink alcohol to participate in this study, we ask about alcohol use only of those women who drank in the past 30 days and administer a series of questionnaires regarding reasons for not drinking and general coping to nondrinkers to similarly balance for time.

The measures in the 4 different EMA surveys (morning, before bed, after binge eating, and prompted) are described in Table 2. Items were either drawn directly from existing EMA scales or past studies or were adapted from non-EMA measures and developed for the purposes of this study in instances where no EMA measure of a construct existed.

The end-of-study survey collects information about participants’ experiences in the study, interest in and willingness to be involved in future mobile health interventions regarding physical and mental health, and COVID-19–related questions. Table 3 provides a description of the constructs included in the end-of-study survey.

**Table 1:** Baseline measures.

Construct and description			Measure name
Demographics—age, sexual orientation (identity, attraction, and behavior), height, weight, address, geographic location, relationship status and length, employment status, education level, average individual income, and finances			N/A^a^
Sexual identity—self-identity, identity disclosure, and “coming out” (for sexual minority women only)			N/A
**Eating and body image**			
	Eating pathology (body dissatisfaction, binge eating, cognitive restraint, purging, restricting, and excessive exercise)	Eating Pathology Symptoms Inventory [57]	
	Diagnostic criteria for eating disorder behaviors	Eating Disorder Examination Questionnaire 6.0—selected items [58]	
	Trait-level body dissatisfaction	Body Shape Questionnaire [59]	
	Societal and interpersonal norms and pressures regarding appearance and norm internalization	Sociocultural Attitudes Toward Appearance Questionnaire-4-Revised [60]	
	Trait-level ability to eat intuitively following their physical hunger and satiety cues	Intuitive Eating Scale-2 [61]	
	Fat talk engagement and negative body-related conversations	Fat Talk Questionnaire [62]	
	Extent to which individuals who engage in disordered eating lie about such behaviors	Deliberate Denial of Disordered Eating Behaviors Scale [63]	
**Gender experiences and identity**			
	Experiences of unfair treatment	Everyday Discrimination Scale [64]	
	Degree to which an individual identifies with and has interest in their own race or ethnicity	Multigroup Ethnic Identity Measure [65]	
	Degree to which people apply weight-based stereotypes to themselves and base their self-evaluations on weight	Modified Weight Bias Internalization Scale [66]	
**Sexual minority women’s experiences, identity, and support** **(for sexual minority women only)**			
	Sexual minority discrimination	Heterosexist Harassment Rejection, and Discrimination Scale [67]	
	Sexual minority identity and psychosocial functioning	Lesbian, Gay, and Bisexual Identity Scale [68]	
	Connectedness to the LGBT^b^ community	Connectedness to the LGBT Community Scale [69,70]	
	Feelings about being part of the sexual minority community and the extent to which their status is important to their identity	Sexual Minority Community Identification [71,72]	
**Women’s identity and support** **(for heterosexual women only)**			
	Feminist identity development	Feminist Identity Composite [73-75]	
	Individuals’ appraisals of perceived social support	Social Support Appraisals Scale [76]	
**Mood, stress, and other general life experiences**			
	Depressive symptoms	Center for Epidemiological Studies Depression 10-item Scale [77]	
	Anxiety symptoms	Generalized Anxiety Disorder 7-item Scale [78]	
	Perceived life stress	Perceived Stress Scale [79]	
	Self-harm and suicidal ideation, plans, and attempts within the past 12 months	Self-Harm Questions-Adapted [80,81]	
	Ability to bounce back or recover from stress	Brief Resilience Scale [82]	
	Engagement in comparison behaviors	Iowa-Netherlands Comparison Orientation Measure [83]	
	Early traumatic experiences and adverse life events	Childhood Traumatic Events Scale [84]	
Past 30-day alcohol use—any alcohol use in the last 30 days			N/A
**Alcohol use** **(for women who drank in the last 30 days)**			
	Quantity, volume, and frequency during a typical week	Daily Drinking Questionnaire [85]	
	Motives for drinking	Drinking Motives Questionnaire [86]	
	Alcohol consequences experienced by young adult drinkers	Brief Young Adult Alcohol Consequences Questionnaire [87]	
	Eating-related behaviors related to alcohol consumption	Compensatory Eating and Behaviors in Response to Alcohol Consumption Scale [88,89]	
**General coping with stress** **(for women who did not drink in the last 30 days)**			
	Importance of various reasons for not drinking	Reasons for Not Drinking scale [90]	
	Strategies for effective and ineffective coping	Brief Coping Orientation to Problems Experienced [91,92]	
**Other health and health behaviors**			
	Health-related quality of life	Medical Outcomes Study Short Form-36 [93]	
	Typical marijuana quantity used each day	General Marijuana Use [85,94]	
	Typical cigarette quantity used each day	General Cigarette Use [85]	
	Perceived drinking norms for heterosexual and sexual minority women	Alcohol Descriptive Norms [95]	
	Reasons why people do not engage in as much physical activity as they think they should	Barriers to Being Active Quiz [96]	
	Expected outcomes of engaging in physical activity	Exercise Motivation Scale [97]	

^a^N/A: not applicable (no named measure).

^b^LGBT: lesbian, gay, bisexual, and transgender.

**Table 2:** Ecological momentary assessment surveys.

Survey, construct, and description				Measure name and reference
**Morning survey**				
	Sleep—time in bed, time to fall asleep, wake-up time, length of time asleep, and quality of sleep		Items adapted from the Pittsburgh Sleep Quality Index [98]	
	Alcohol use—quantity, time drinking, type, location, and interactions (for drinking days only)		Items adapted from various sources, including the study by Heron et al [99]	
	Compensatory behaviors for drinking (for drinking days only)		Items adapted from the Compensatory Eating and Behaviors in Response to Alcohol Consumption Scale [88]	
	Nondrinking questions (for nondrinking days only)		Items adapted from various sources, including the study by Heron et al [99]	
	Mood—anticipated mood for the upcoming day: happy, bored, relaxed, sad, excited, content, worried or anxious, angry, frustrated, or energetic		Items adapted from the Positive and Negative Affect Schedule-Expanded Form [100,101]	
	Anticipated upcoming stressful and pleasant experiences		Items developed for this study	
**Before-bed survey**				
	Mood—overall mood for the day: happy, bored, relaxed, sad, excited, content, worried or anxious, angry, frustrated, or energetic		Items adapted from the Positive and Negative Affect Schedule-Expanded Form [100,101]	
	Social comparison—nature of social comparison, number of times, type of comparison, person compared with, and feelings about comparison (for social comparison days only)		Items were adapted from sources including the study by Arigo et al [102]	
	General social experiences—types of people interacted with, pleasantness of interactions, and importance of interactions and topics (for non–social comparison days only)		Items developed for this study	
	Tobacco—tobacco use		Items developed for this study	
	Physical activity—length, type, amount, thoughts about activity, estimated amount of physical activity, and exercise the following day		Items adapted from multiple sources [102,103]	
**Post–binge eating survey**				
	Date, time, and location of binge eating		Items developed for this study	
	Eating speed, fullness, with others, and emotions after eating		Items adapted from the Eating Disorder Diagnostic Scale [104] and the DSM-5^a^ binge eating description [105]	
	Purging and intended purging		Items adapted from the Eating Disorder Diagnostic Scale [104]	
	Mood before, during, and after binge eating		Items developed for this study based on pilot study participant feedback	
	Perceived factors contributing to the binge eating episode		Items developed for this study based on pilot study participant feedback	
**Prompted survey**				
	Location activity—location of survey notification and activity upon notification		Items developed for this study	
	Mood—current mood ratings for happy, bored, relaxed, sad, excited, content, worried or anxious, angry, frustrated, or energetic		Affect words selected from the Positive and Negative Affect Schedule-Expanded Form [100,101]	
	Body image—satisfaction with physical appearance, body shape, weight, physical attractiveness, and looks		Items from the Body Image States Scale [106] adapted from a 9-point scale to a 7-point scale [103]	
	Self-objectification—thinking about appearance to other people		Item adapted from the study by Holland et al [107]	
	General stress—stressful or unpleasant experiences		Items adapted from the Daily Inventory of Stressful Events [108] and used in the study by Heron et al [109]	
	Identity stress—discrimination based on aspect of identity and aspect of identity responsible for discrimination		Items adapted from the study by Panza et al [110]	
	Disordered eating behaviors—overeating, loss of control of eating, guilt after eating, emotional eating, concerns about eating with others, restriction, avoiding foods		Items from the Eating Disorder Examination Questionnaire 6.0 [111], items adapted using language from the DSM-5 [105], and items from the Emotional Eater Questionnaire [112]	
	Intuitive eating behaviors—eating for physical hunger, trusting body to eat, body-food choice congruence, permission to eat desirable foods		Items adapted from the Intuitive Eating Scale [61,113]	
	Physical activity—intensity level of physical activity, type of exercise, length of exercise, and typical amount of exercise		Items adapted from the International Physical Activity Questionnaires [114]	
	Media use—social media use, type, and activity		Items developed for this study	
	Appearance-related pressures and source of pressures		Items adapted from the Sociocultural Attitudes Toward Appearance Questionnaire-4 [115]	
	Negative body-related talk and source of talk		Items adapted from the Fat Talk Questionnaire [62]	
	Social interactions—type, pleasantness, and importance of people and topic of interaction		Items adapted from the studies by Bernstein et al [116] and Zhaoyan et al [117]	

^a^DSM-5: Diagnostic and Statistical Manual of Mental Disorders, Fifth Edition.

**Table 3:** End-of-study survey.

Construct and description			Measure name
Accessibility of EMA^a^ surveys—questions measuring the burden and accessibility of the EMA surveys			Items developed for this study
Experiences with post–binge eating surveys—measured participants’ accurate identification of binge eating episodes and feedback on the survey			Items developed for this study
Identity inclusivity feedback—assessed whether survey items were inclusive of participant identities and captured their experiences			Items developed for this study
Desire to improve health—measured desire and interest in improving physical and mental health			Items developed for this study
Help seeking—assessed resources in social circle that could help the participant address health problems (assessed physical health and mental health problems separately)			Adapted from the General Help-Seeking Questionnaire [118]
Willingness to use mobile health technology—assessed willingness to use mobile health technology to improve physical and mental health or implement health behavior changes			Adapted from the study by Cramer et al [119]
COVID-19 situation—current and previous state of shelter-in-place orders, social distancing practices, and experience of illness with COVID-19			Items developed for this study
COVID-19 health impacts—impact of the COVID-19 pandemic on physical and mental health symptoms as well as change in health behaviors since the start of the pandemic			Items developed for this study [120]
COVID-19 general impacts—changes in responsibilities, finances, and work or school roles since the start of the COVID-19 pandemic			Items selected from the Epidemic-Pandemic Impacts Inventory [121]
COVID-19 minority impacts—impact of the COVID-19 pandemic on minority experiences			Open-ended questions developed for this study

^a^EMA: ecological momentary assessment.

### Data Analysis Plan

EMA data are inherently hierarchical, with surveys (*level 1*) nested within a person (*level 2*). As such, we will use multilevel modeling to account for similarities between responses for each person. Before hypothesis testing, the data will be examined for normality, outliers, and nonlinear relationships between continuous variables. Model comparison procedures will be used to determine the fixed versus random effects for each model.

Multilevel modeling is robust to missing data (eg, skipped EMA surveys). For variables skipped within a completed assessment, expectation maximization imputation will be used to replace missing values, an approach demonstrated to minimize bias [122]. Participants with complete data will be compared with those with missing data to identify potential attrition biases, and significant predictors will be included as covariates in all analyses.

To test our main hypotheses, level-1 associations between relevant predictors (negative emotions, social factors, and health behaviors) and outcomes (binge eating and loss of control of eating) will be examined using a series of multilevel models: isolated models with each predictor modeled separately first and then a final model examining all predictors simultaneously to explore unique effects (hypotheses 1a-b). Sexual identity will then be included as a level-2 predictor, and cross-level interactions between sexual orientation (*level 2*) and general factors (*level 1*) will indicate whether momentary associations are different across groups (hypothesis 1c). Aim 2 analyses will use only sexual minority women’s data. Level-1 associations between sexual minority stress and eating outcomes will be examined using the same approach as in aim 1: a series of isolated then simultaneous multilevel models (hypothesis 2a). To assess hypothesis 2b, a multilevel structural equation model will be used to examine if negative affect mediates these relationships (1-1-1 model) [123] using Monte Carlo CIs to assess the significance of indirect effects [124]. For aims 1 and 2, we will conduct the aforementioned analyses for momentary associations (random surveys) and event-lagged associations (event and random surveys), allowing for the examination of time-lagged associations (eg, negative affect *before* the event). Aim 3 analyses will examine race- and person-level body- and eating-related factors (BMI, body dissatisfaction, disordered eating, and thin norm internalization), and sexual minority–specific factors (lifetime discrimination, positive sexual minority identity, and community identification and connection) will be included as level-2 predictors in a series of models. Cross-level interactions will indicate whether person-level factors moderate level-1 associations between general factors and binge eating (moderation of aim 1) or between minority-specific factors and binge eating (moderation of aim 2). Three-way interactions among level-1, level-2 sexual orientation, and other level-2 moderators will indicate whether the differential associations detected in the second component of aim 1 (ie, whether associations are stronger or weaker across sexual identity) are moderated by other person-level factors.

## Results

Recruitment began in February 2021 and is expected to continue until fall 2022. We have currently consented 129 sexual minority women and 146 heterosexual women into the study. Data cleaning and analysis will begin after data collection is complete.

## Discussion

### Overview

The goal of the HER Life Project is to examine general and sexual minority–specific predictors of disordered eating, in particular binge eating, among sexual minority and heterosexual young women in their daily lives. This study uses an EMA approach to assess participants at fixed times (morning and before bed), at semirandom times throughout the day, and following self-reported binge eating episodes, thus providing rich information regarding the affective, social, behavioral, and minority-specific experiences of these women. Although there is a growing body of literature considering real-world associations among affective processes (eg, negative affect, stress, and body dissatisfaction), social processes (eg, interactions and social comparisons), and binge eating in general samples of young women where sexual identity is not known or reported, it remains unclear whether and how these processes operate for sexual minority women and if there are minority-specific predictors (eg, sexual minority stressors) for these women as well. This study includes samples of both sexual minority and heterosexual young women in an effort to explore potential similarities and differences in binge eating experiences and predictors. We expect that there will be many similar affective, social, and health behavior predictors of binge eating across sexual minority and heterosexual women but that there may also be some differences, particularly with regard to body dissatisfaction and unique sexual minority stressors. The findings from this study can help improve our understanding of potential processes that may contribute to health disparities between sexual minority and heterosexual young women’s disordered eating behaviors and can be used to inform culturally tailored interventions for binge eating for young sexual minority women. In the following sections, we discuss some of the questions we considered and the challenges we have experienced when designing and carrying out the HER Life Project.

### Methodological Challenges

#### Defining Sexual Identity and Gender Identity

One of the ongoing challenges we experience in our work with sexual minority women is identifying the optimal way to define “sexual minority” and operationalize our definition during the screening process. We initially planned to recruit participants who identified as lesbian women. However, based on some of our previous work [99,125] and in consultation with Community Marketing & Insights, the market research firm we worked with that specializes in recruiting LGBTQ+ adults for web-based research, we learned that young women are increasingly choosing to use other labels when describing their sexual identity (eg, queer or pansexual), use multiple labels (eg, lesbian and queer or gay and queer), or are resisting labels completely [126,127]. Therefore, from the start of this study, we decided to instead focus on recruiting based primarily on sexual attraction and, in some cases, relying also on behavior (ie, gender of the most recent romantic partner). We used a single attraction question that asked people to describe who they were attracted to, with response options including “only or mostly attracted to women,” “only or mostly attracted to men,” “equally attracted to men and women,” or “attracted to people regardless of their gender identity” (“other” and “prefer not to answer” responses were also available). Potential participants were eligible for the heterosexual group if they selected “only or mostly attracted to men.” Potential participants were eligible for the sexual minority group if they selected “only or mostly attracted to women” or if they selected “equally attracted” or “attracted regardless of gender” and also endorsed that their current or most recent relationship was with a woman. An ongoing challenge in research on sexual minority individuals is to balance our desire to be inclusive and respectful of the many ways in which identity (including multiple identities) can be defined against our desire to maximize study validity. A sample that is too heterogeneous in terms of identity creates challenges in applying previous literature to our work and interpreting our findings. Therefore, we settled on these criteria in an effort to be more broadly inclusive of sexual minority women who have diverse attractions (ie, including those with attractions to multiple genders or regardless of gender) but also recognize that, because of the focus in this study on body image and eating behaviors, in the sexual minority women group, we wanted to enroll people who either were attracted to women or were currently or had been recently romantically involved with a woman.

In addition to the challenges in describing sexual identity, we also considered how we defined gender. The focus of this study was on women, and we decided to restrict the sample to people who reported that they were assigned female at birth. We also inquired about potential participants’ gender identity, and our initial plan was to only include individuals who identified as “women.” However, again in consultation with Community Marketing & Insights, we learned that they are increasingly seeing people identifying with more than one gender label, particularly nonbinary or gender nonconforming. Thus, for this study, we included participants who selected “woman” only or selected both “woman” and either “gender queer/nonconforming” or “nonbinary.” Overall, when considering our inclusion criteria for sexual minority and heterosexual women, we tried to balance recruiting relatively homogeneous samples that were distinct from each other based on sexual attraction while also being inclusive of the diverse ways in which young adults describe and understand their identities and attractions [128-130]. These issues of how to define and describe sexual minority individuals will likely be an ongoing challenge for researchers and will continue to evolve as language and cultural understanding change over time.

#### Remote Data Collection Considerations

Given the large number of young women with diverse sexual identities that we are recruiting for this study, we planned for national recruitment across the United States, which requires that all data collection occur entirely remotely. From the start of the study, we implemented several strategies to enhance study engagement and compliance and also added new protocols throughout based on our experiences. First, to better describe the study to potential participants, we developed several training videos that described the study very generally and others that provided more specific details regarding the study procedures and explained study surveys and materials (eg, defining binge eating). These materials are distributed via email before and during the consent process and are embedded within Qualtrics (Qualtrics International Inc) surveys, allowing us to record whether and for how long the participants review the videos. Participants also have access to several of these relevant videos on the survey app on their phones and can review them throughout the study as needed. On the basis of our pilot work, these videos were well received, and pilot participants described them as useful in orienting them to the intensity of the study procedures so they had a better idea of what to expect when participating.

Second, as part of the screening process, participants shared with us whether they had an Android or Apple phone that they would be willing to use for the study. As a way to confirm potential participants’ access to an appropriate smartphone, as part of the screening process (before consent), we ask participants to download the app to their phones. Although we cannot objectively confirm that they do so, we provide step-by-step downloading instructions as part of a Qualtrics survey and, at the end of the survey, participants indicate that they completed the installation. If participants are unable to install the app on their own phone or do not have a phone that the app is compatible with, we have phones that can be mailed to participants to use for the duration of the study. However, to date, all potential participants have had their own phones to use for the study. This is not surprising given that, in 2021, it was estimated that 96% of adults aged 18 to 29 years owned a smartphone [131]. Together, this suggests that future studies of young adults can expect that most if not all participants will have smartphones that are appropriate for a survey-based EMA study such as this one.

A third procedural consideration that has evolved over the course of the study is how we handle moving people through the study procedure remotely. As described in the Methods section, we developed a reminder system from the start of the study, which includes sending up to 2 reminders, typically several days apart, to participants who are not progressing through the study. Although most participants require very few or no reminders, for those who do receive all the planned reminders from our study team, before either withdrawing them from the study (if they previously consented) or assuming that they are not interested in enrolling (if before consent), the PI sends a final email inquiring about their interest in continuing. Although the PI email does not always result in re-engagement in the study, there have been many instances where it has helped bring a participant back to the study. Overall, this general approach of reminders is helping participants move efficiently through the study process while our study team primarily interacts with participants via email.

The onset of the COVID-19 pandemic and the associated safety protocols and guidelines limited face-to-face interactions in research settings. Given that our study onboarding and data collection procedures were designed to be remote, we experienced a rather seamless transition to conducting our research in the postpandemic era with little to no interruption. Our experiences, coupled with the likelihood of most young adults having a smartphone appropriate for survey-based EMA studies, suggest that entirely remote onboarding and data collection procedures are feasible for national recruitment and in other situations where bringing participants into the laboratory is not always feasible or possible.

### Future Research Directions and Study Implications

The HER Life Project primarily focuses on exploring potential similarities and differences in predictors of binge eating between sexual minority and heterosexual young women given the documented health disparities in obesity rates and binge eating between these groups. However, as described in this protocol, part of aim 3 is to explore potential disparities that Black women may experience and consider how racial and sexual minority identities may intersect for Black sexual minority women. As described previously, there is very limited previous research on disordered eating among people who identify as both racial and sexual minority women [46] and, thus, this is a critically understudied area. Recent reports have highlighted the need for more research using an intersectional perspective and reiterated the importance of such work for providing a more complete understanding of minority health more broadly. Although this study will provide some insight on these topics, it is only a first step, and future work to evaluate the role of other identities (eg, other racial or ethnic group identities, location [urban or rural], and socioeconomic status) and their interaction with sexual orientation is needed to advance the health and well-being of all sexual minority individuals [132]. Continuing to expand and incorporate an intersectional framework will enrich both research and intervention, with ultimate benefits to education, health care, and public policy.

Finally, we view the HER Life Project as 1 step in a large research program aimed at developing culturally sensitive interventions to address binge eating and obesity in sexual minority women. Data from this study can help inform future work in 2 ways. First, as we are including both sexual minority and heterosexual young women, this study will allow us to determine whether there are differential associations between the hypothesized predictors and binge eating or if sexual minority–specific factors (eg, experiencing sexual minority stressors and discrimination) are associated with binge eating. These findings could help in identifying intervention content for disordered eating or body image interventions that require tailoring for sexual minority women. For example, it is plausible that intervention components teaching strategies for coping with sexual minority stress (instead of engaging in binge eating) are needed. The HER Life Project is also uniquely designed to inform the development of ecological momentary interventions (EMIs). Binge eating EMIs that use mobile apps or SMS text messaging can deliver treatment in women’s everyday lives and at specific times when most in need (ie, when likely to engage in binge eating) [133,134]. To develop EMIs, EMA data regarding within-person associations *with the target population* are first needed [135-137], and this study provides such data. In summary, the HER Life Project will provide foundational information needed to develop culturally tailored treatments and mobile technology–based interventions for sexual minority women with the ultimate goal of reducing binge eating and obesity disparities.

## Appendix

Multimedia Appendix 1 Peer-review report from the Psychosocial Risk and Disease Prevention (PRDP) Study, Section - Risk, Prevention and Health Behavior Integrated Review, Group - Center for Scientific Review (National Institutes of Health, USA).

## Abbreviations

EMA: ecological momentary assessment
EMI: ecological momentary intervention
HER Life: Health and Experiences in Real Life
ICC: intraclass correlation coefficient
LGBTQ+: lesbian, gay, bisexual, transgender, and queer
PI: principal investigator

## Data Availability

Data will be available from the principal investigator upon reasonable request.
