# Supplementary material for: Rationale and Design of an Ecological Momentary Assessment Study Examining Predictors of Binge Eating Among Sexual Minority and Heterosexual Young Women: Protocol for the Health and Experiences in Real Life (HER Life) Study
Source: JMIR Res Protoc. 2022 Oct 21;11(10):e41199. doi: 10.2196/41199 (PMC9636528; doi:10.2196/41199)
Supplement: Multimedia Appendix 1 [file resprot_v11i10e41199_app1.pdf]

SUMMARY STATEMENT
(Privileged Communication)

PROGRAM CONTACT:
Meryl Sufian
301-594-8758
sufianm@mail.nih.gov

Release Date: 06/22/2018
Revised Date:

Application Number: 1 R01 MD012598-01A1

Principal Investigator

HERON, KRISTIN E

Applicant Organization: OLD DOMINION UNIVERSITY

Review Group: PRDP
Psychosocial Risk and Disease Prevention Study Section

Meeting Date: 06/11/2018
Council: OCT 2018
Requested Start: 09/01/2018
RFA/PA: PA18-037
PCC: IBB02MSU
Dual IC(s): MH

Project Title: Examining binge eating in daily life: Working toward reducing obesity disparities in racially diverse lesbian and heterosexual women
SRG Action: Impact Score:31 Percentile:17
Next Steps: Visit https://grants.nih.gov/grants/next_steps.htm
Human Subjects: 30-Human subjects involved - Certified, no SRG concerns
Animal Subjects: 10-No live vertebrate animals involved for competing appl.
Gender: 2A-Only women, scientifically acceptable
Minority: 1A-Minorities and non-minorities, scientifically acceptable
Children: 3A-No children included, scientifically acceptable

Project Year	Direct Costs Requested	Estimated Total Cost
1	150,000	228,824
2	175,000	266,961
3	200,000	305,098
4	175,000	266,961
TOTAL	700,000	1,067,843

ADMINISTRATIVE BUDGET NOTE: The budget shown is the requested budget and has not been adjusted to reflect any recommendations made by reviewers. If an award is planned, the costs will be calculated by Institute grants management staff based on the recommendations outlined below in the COMMITTEE BUDGET RECOMMENDATIONS section.
NEW INVESTIGATOR

1R01MD012598-01A1 HERON, KRISTIN

COMMITTEE BUDGET RECOMMENDATIONS
EARLY STAGE INVESTIGATOR
NEW INVESTIGATOR

RESUME AND SUMMARY OF DISCUSSION: This application seeks support to conduct an ecological momentary assessment (EMA) study to understand the relationship of stress and affect in relationships among racially diverse lesbian and heterosexual women and their association to binge eating (BE). This resubmitted work was highly responsive to previous critiques resulting in work that the review panel opined offers high impact to further scientific understanding of BE additionally illuminating health disparities. Reviewers again noted many strengths: The scientific premise is excellent, focusing on the well-known issue that stress is a major factor precipitating BE, and little is known about BE patterns of black and lesbian women. The outstanding Principal Investigator is complemented by a well-qualified team that offers complementary expertise further strengthened by an outstanding research environment. The innovation is deemed outstanding with the focus on affective and social factors and health behaviors (alcohol use and physical activity) associated with BE in natural settings, use of mobile technology based EMA methods, and inclusion of unique sexual minority-specific factors inherent in the study population of lesbians and minorities with BE. The scientific rigor is very good, strengthened by alcohol and physical activity measurements, strong preliminary studies, and use of EMA methods. Some moderate and minor weaknesses were discussed. Of moderate concern, 1) the reviewers noted that the discussion of black versus white study sample lacked clarity in that it was unclear if the non-blacks were white and ultimately the power to discern racial differences was insufficient as was the recruitment strategy, and 2) the definition of BE was inaccurate leading to an overestimate of BE which could weaken the study, limit recruitment and lessen study feasibility. And minor issues included a timeline considered too long and unjustified for continued pilot testing, the sexual minority stress questionnaire lacked specifics and didn't discuss test-retest reliability, the physical activity assessment was insufficiently detailed and the 18-30 age range was considered possibly too broad, introducing generational and developmental differences that could weaken study associations. In sum, reviewers remain very enthusiastic about this potentially high impact work that offers notable strengths to reduce obesity disparities but a few remaining weaknesses limit the highest potential of this work.

DESCRIPTION (provided by applicant): Young adult lesbian women are twice as likely to be overweight and obese as their heterosexual peers and Black women are similarly more likely to be obese than White women. These minority women are thus more prone to obesity-related comorbidities including heart disease, stroke, and type 2 diabetes, which are leading causes of death in the U.S. Binge eating behaviors (overeating, loss of control of eating) contribute to obesity and are particularly prevalent in young adult women. Despite well documented disparities in binge eating, little is known about contributing factors in lesbian women, and in particular racial minorities. In studies of young women where sexual orientation is not known, assessed, or reported (hereafter referred to as general samples), research demonstrates binge eating is associated with affective states, social processes, and health behaviors. Studies by our group and others using mobile technology-based ecological momentary assessment (EMA) have examined how daily affective and social experiences influence young women's eating in everyday life. These studies were conducted with general samples of primarily White women; how affective states, social processes, and health behaviors in daily life impact lesbian women's binge eating remains unclear. Consistent with minority stress theories, preliminary data also suggest sexual minority stress – or the stress people from stigmatized groups are exposed to due to their marginalized social status – likely influences binge eating in lesbian women, but the role of unique minority experiences in binge eating has not been examined using EMA. Race, eating-related factors, and sexual minority-specific factors may also moderate daily associations, but have not been fully explored. In particular, implications of having intersecting sexual- and racial-minority identities (i.e.,

being a Black lesbian women) on binge eating have yet to be considered. To address these limitations in the binge eating and sexual minority literatures, the proposed study examines affective, social, health behavior, and sexual minority-specific factors associated with binge eating in natural settings. Young adult lesbian (n=150, 50 Black) and heterosexual (n=150, 50 Black) women ages 18-30 who engage in binge eating behavior will complete brief smartphone-based EMA surveys five times daily and in response to binge behaviors for two weeks. Study aims include examining how daily affective, social, and health behavior factors impact lesbian women's eating, and how sexual minority-specific experiences uniquely contribute to binge eating in daily life. We will also explore how race moderates these associations. The EMA design allows examination of daily processes in natural settings, and including racially diverse lesbian and heterosexual women allows identification of factors that contribute to disparities. This study fills research gaps by expanding understanding of general and sexual minority-specific factors that contribute to binge eating in daily life, and the role of race in these associations. Such information is critical for informing the development of culturally tailored interventions for lesbian women, with the ultimate goal of reducing binge eating and obesity disparities.

PUBLIC HEALTH RELEVANCE: Young adult lesbian women and Black women are at greater risk for obesity and binge eating than their heterosexual and White peers, yet relatively little is known about factors contributing to these disparities. The proposed research uses ecological momentary assessment (EMA) to examine associations between affective, social, health behavior, and sexual minority-specific factors and binge eating in the daily lives of racially diverse young adult lesbian and heterosexual women. Such information is critical for informing the development of culturally tailored interventions for lesbian women, with the ultimate goal of reducing binge eating and obesity disparities.

CRITIQUE 1

Significance: 3
Investigator(s): 1
Innovation: 1
Approach: 7
Environment: 1

Overall Impact: This revised and resubmitted application proposes a study of affective, social, health behavior and sexual minority specific factors associated with binge eating in adult lesbian (n = 150) and heterosexual (n = 150) women, using mobile technology based daily ecological momentary assessment (EMA) over a two-week period. Most of the limitations noted in prior reviews have been addressed. The application has been highly responsive to previous reviews which had noted concerns about scientific premise and rigor. The scientific premise is clearly articulated in a critical literature review and supported by preliminary data from the Principal Investigator's work. Public health significance is high in that the proposed study addresses an important health risk behavior (binge eating) in an under researched population (sexual minority women). Significance further derives from the inclusion of a large subsample of black women, a population group thus far largely absent from EMA studies of eating behavior. The investigative team is well qualified and the resources are excellent. Scientific rigor is high with one noted major and a couple of minor exceptions. Major concerns arise from the definition and measurement of binge eating which may limit the generalizability of findings and make it difficult to compare findings of the proposed study with those published already. A minor concern is the still relatively unspecified reliability of the measure of sexual minority specific stresses and lack of detail about the physical activity measure. Enthusiasm for the overall research is very high; yet due to concerns about the approach the overall impact is estimated to be moderate.

1. Significance:

Strengths

- Overweight/obesity are elevated in women from sexual minority groups and in ethnic minority women. Weight disorders represent a significant public health concern.
- The Institute of Medicine has identified health disparities between sexual minority women and heterosexual women a research priority.
- Extensive research supports concurrent and prospective associations between binge eating and weight gain, as well as between binge eating and overweight/obesity in studies of mostly heterosexual women or women of unknown sexual orientation.
- The application articulates a clear and compelling theoretical rationale for the study aims and approach.
- Recent studies have begun to explore whether overeating, loss of control eating, or binge eating (which is defined as overeating accompanied by a sense of loss of control) have differential associations with health correlates or outcomes such as overweight/obesity and other health risk behaviors (e.g., alcohol use).
- The addition of measures of alcohol use and physical activity enhance the scientific premise.

Weaknesses

- Although the Principal Investigator has been responsive to prior critiques by now explicitly focusing on binge eating, the definition of binge eating is problematic in that it does not map on to commonly used definitions. The application focuses on “binge eating behaviors” defined as “overeating” or “loss of control eating”; the application is silent on the relationship between those two behaviors (see also approach). The idiosyncratic use of the term “binge eating behaviors” limits the significance of the proposed research (moderate concerns).

2. Investigator(s):

Strengths

- The Principal Investigator, Dr. Kristin Heron is a NI/EDI and Assistant Professor of Psychology at Old Dominion University. Her research focuses on disordered eating and body image concerns. She has extensive experience with EMA.
- The co-investigative team adds expertise in sexual minority stress and health (Dr. Lewis) and multilevel/hierarchical modeling (Braitman).

Weaknesses

- None noted.

3. Innovation:

Strengths

- Theories of sexual minority stress and health have not yet been tests as applicable to binge eating.
- The application of EMA to examine binge eating in sexual minority women is innovative.
- The examination of the intersection of sexual minority status and race is innovative
- The focus on three health behaviors within the EMA paradigm, namely binge eating, alcohol use, and physical activity, is innovative.

Weaknesses

- None noted.

4. Approach:

Strengths

- Clear description of how the study sample will be recruited and recruitment of a sample across the United States is a definite strength.
- Age group to be included (18 to 30) is well-justified.
- Theory-driven selection of study variables/measures.
- Analysis plan accounts for missing values and multiple comparison issues.
- Extensive pilot data suggest feasibility.
- The investigators have extensive experience with EMA methods.
- The Principal Investigator has been responsive to prior critiques about the sampling schedule for EMA and now will use a combination of random time sampling and event sampling.
- The application now focuses on binge eating, as suggested in prior reviews (see, however, weaknesses).
- Responding to prior critiques, the application now specifies collection of additional health variables with strong scientific premise for inclusion, namely physical activity and alcohol use.
- The analysis plan is well-connected to the study aims and addresses issues such as missing data.

Weaknesses

- Key weakness is the conceptual and methodological approach to binge eating (Moderate concerns). Specifically:
- Definition of binge eating is idiosyncratic; the term binge eating is used interchangeably with the term "binge eating behaviors". It is unclear why the widely-accepted definition of binge eating is not applied in the proposed research (binge eating, by definition, involves overeating accompanied by a sense of loss of control over the eating episode). Although the investigators will be able to measure binge eating (defined as overeating with loss of control), the absence of any discussion of the possible differences in associations between binge eating versus loss of control eating or overeating and study variables of interest (e.g., stress) is worrisome.
- Conceptually and clinically, "overeating" and" loss of control eating" are not synonymous. Prior EMA studies have shown different temporal trajectories of negative affect for "binge eating" versus "overeating" versus "loss of control eating in the absence of overeating." Clarity concerning the definitions and measurement of "binge eating behaviors" is needed.
- Inclusion criteria specify at least two episodes of "overeating or loss of control eating" in the past two weeks. Since these behaviors will be measured using the Eating Disorder Examination-Questionnaire (EDE-Q), an instrument that focuses on the past 28 days, it is unclear how it will be determined that at least two episodes occurred in the past 14 days. Moreover, unless the EDE-Q is modified, in its original form it does not measure overeating and loss of control eating separately; rather, loss of control is asked only when overeating has been reported.
- A case could be made for measuring overeating, loss of control eating in the absence of overeating, and binge eating. What would be important, however, is to consider the impact of using an "or" algorithm for recruitment, because there may be demographic differences in the prevalence of these behaviors. Therefore, it is possible that the "or" algorithm (used in the inclusion criteria) will result in differential distribution of overeating versus loss of control eating in the different demographic groups. If this were to be the case, it would become difficult to

disentangle variables such as race as a possible moderator of the relationship between “binge eating behaviors” and weight.

- The estimated prevalence of “binge eating behaviors” appears unrealistically high (~17%) both in regard to epidemiological studies of binge eating and in regard to the investigator’s prior research. Given the varying approaches to the definition of binge eating in the Principal Investigators published work it is unclear on what definition/measure the expected prevalence is based.
- The Principal Investigator now reports Cronbach’s alpha (internal consistency) for an 8-item daily sexual minority stress measure; it remains unclear whether the instrument is an accurate and valid measure.
- The physical activity measure was used in previous work by the Principal Investigator; the previous studies were presented at conferences, making it difficult to discern the actual items, the feasibility and the reliability of these measures in the proposed work.

5. Environment:

Strengths

- The environment is fully adequate for successful execution of this study.

Weaknesses

- None noted.

Study Timeline:

Strengths

- A detailed timeline is provided; the overall duration of the study is not adequately justified seems unduly long for achieving the study aims.

Weaknesses

- None noted.

Protections for Human Subjects:

Acceptable Risks and/or Adequate Protections

- This is a low risk study with adequate protections of data privacy. Participants will receive information about resources for eating disorders.

Data and Safety Monitoring Plan (Applicable for Clinical Trials Only):

Not Applicable (No Clinical Trials)

Inclusion of Women, Minorities and Children:

- Sex/Gender: Distribution justified scientifically
- Race/Ethnicity: Distribution justified scientifically
- For NIH-Defined Phase III trials, Plans for valid design and analysis: Not applicable
- Inclusion/Exclusion of Children under 18: Excluding ages <18; justified scientifically
- The study includes women only; this is scientifically justified based on extensive prior research documenting that the experiences of sexual minority groups vary by gender.

- Oversampling of black women will ensure that race can be examined as a moderator variable.
- The exclusion of children is justified based on epidemiological studies demonstrating that binge eating is relatively uncommon in children; the proposed study will capture population group at highest risk for reporting binge eating.

Vertebrate Animals:

Not Applicable (No Vertebrate Animals)

Biohazards:

Not Applicable (No Biohazards)

Resubmission:

- The investigator has been highly responsive to several key concerns expressed in prior critiques. One major change is the focus in the revised application on binge eating. Unfortunately, although the investigator has made an effort to respond, the specific response raises new concerns due to the unusual operationalization of binge eating. The revised application now also proposes to measure other key health behaviors and this has strengthened the application. In the revised application, methodological details have been clarified.

Budget and Period of Support:

Budget Modifications Recommended (in amount/time)

Recommended budget modifications or possible overlap identified:

- In prior reviews, the timeline was found to be excessively long. Although the investigator has reduced the time frame from 5 to 4 years, the proposed timeline still seems overly long and poorly justified. For example, 15 months of start-up time seems excessive in light of the investigator's description of having piloted measures and approach. As was previously suggested, the project could be reduced to 2 to 3 years.

CRITIQUE 2

Significance: 3
Investigator(s): 2
Innovation: 3
Approach: 3
Environment: 2

Overall Impact: This is a resubmission of an application by a new investigator which seeks to examine the association between negative affect, social interactions, sexual minority status stress, and health behaviors in the occurrence of binge eating behavior among young lesbian and heterosexual women, with an additional focus on the intersection between race and sexual minority status in this context. Strengths of the application include high significance in identifying factors driving health disparities related to obesity in lesbian women, strong scientific premise for several aspects of the proposed work, a cohesive investigator team, and several rigorous elements of the design (partnership with a firm well-versed in recruiting lesbian women, daily and event-based surveys, overall analytic plan). The Principal Investigator and Co-Is have addressed several prior comments adequately, although weaknesses are

noted which reduce enthusiasm slightly, including underdeveloped discussion of how race might intersect with minority sexual status in the context of directionality of increasing or decreasing maladaptive food intake, inadequate consideration for generational and developmental differences in interpreting previous literature on sexual minority status and how these impact current hypotheses, concerns regarding feasibility of recruiting 50 Black lesbians, and inadequate attention to relevant factors – BMI, SES – in the analysis. Overall impact is judged to be moderate to high.

1. Significance:

Strengths

- The prevalence of obesity is significantly increased amongst lesbian (vs. heterosexual) women, predicting an increased rate of comorbid health conditions in this population. Few studies have examined the factors contributing to this increased rate.
- There is a strong scientific premise for the stronger association in women between having a female sexual partner and binge eating, and the use of EMA to assess contributing factors with higher temporal resolution.
- There is modest support from small preliminary studies for the premise that body dissatisfaction is associated with recent food intake, and for the premise relating sexual minority discrimination with greater negative affect and binge eating.

Weaknesses

- It is unclear whether the investigators have adequately accounted for developmental and generational drivers in focusing the aims and hypotheses on women aged 18-30, or acknowledged such factors. It appears that the overarching hypothesis is that sexual minority stress is a more significant driver of binge eating in lesbian women, vs. negative affect, body dissatisfaction, and media exposure in heterosexual women. If this is true, how does that play out in terms of young adults (18-25) vs. women in their late 20s-30s, and older adult women with respect to cumulative effects on body weight, particularly in the context of documented generational differences in sexual minority stress?
- Data supporting clear differences between Black and non-Black lesbian women are not particularly strong. In fact, while among heterosexual women, rates of binge eating frequency appear increased in Black women, the opposite appears true amongst lesbian women. It is unclear whether the current study is adequately designed to address the factors behind these opposing trends.
- In general, preliminary studies are modest and fall short of fully supporting the complex model proposed for differential drivers of binge eating in heterosexual vs. lesbian women, and potential person-level attributes contributing therein.

2. Investigator(s):

Strengths

- The Principal Investigator is a new investigator with a strong background in the development and implementation of EMA/EMI methodology to study eating behaviors in women, including women with sexual minority status.
- Co-I Lewis is a senior faculty member with expertise in sexual minority health and prior success in recruiting the target population through proposed recruitment avenues; Co-I Braitman is a junior faculty member with experience in multilevel modeling.
- The investigator team has a history of collaborative work; two of the investigators are licensed clinical psychologists.

Weaknesses

- None noted

3. Innovation:

Strengths

- This would be the first study to examine the relationship between sexual minority stress, race, and binge eating with higher temporal resolution (minutes to hours) in a diverse sample of lesbians; in that respect, it is innovative.
- Exploring the role of health behaviors (alcohol use, physical activity) in target associations is somewhat novel.

Weaknesses

- The EMA methodology proposed for the project is not particularly innovative.

4. Approach:

Strengths

- Preliminary data support the utility of the EMA-based assessment of sexual minority stress, feasibility of assessing binge eating behavior frequency in young adult women using EMA, and estimates of individuals in the target population who will meet eligibility criteria.
- There are relationships in place with market research firms through which target samples will be recruited.
- Baseline and EMA measures have been carefully chosen and generally appear to be rigorous assessment of behaviors/factors of interest, and appear to be able to complete within a brief amount of time (for the EMA measures).
- The team has piloted the majority of measures, including establishing the psychometric properties of the Social Processes and Sexual Minority Stress measure, which has informed the selection of the frequency of EMA data collection using daily and event-based surveys.
- Methods for minimizing attrition appear to be in place.
- The analytic plan includes rigorous modeling approaches.

Weaknesses

- There are discrepancies between phrasing in the significance section (in which “Black-White” differences are proposed to be examined) and the approach/inclusion enrollment report section (in which the racial breakdown appears to include other racial categories). It is unclear whether the focus is on White vs. Black or Non-Black vs. Black.
- There is a concern regarding feasibility in recruiting the n=50 Black lesbian women from the CMI research panel group. If 15% of the ~2,000 members women in the targeted age range (18-30) are Black, that would mean that with a conservative estimate of 17% meeting binge eating criteria (from Preliminary studies 5a and 5b), which seems to somewhat of an overestimation, there would be n=51 Black lesbian women meeting binge eating criteria. This suggests in order to meet recruitment goals for this group, all of these women would have to be interested, agree to participate, and complete the study. There are no data included supporting the feasibility of this goal.
- It is unclear how the potential confound of group-level differences in BMI will be handled in analysis of Aim 1. If lesbian women have twice the rate of obesity, it seems likely that there will be significant difference in the prevalence of obesity between the lesbian and heterosexual

groups; how will this be handled? It is notable that although the initial premise rests on the association between binge eating and obesity, the study/analytic design does not really address this (beyond using BMI as a “person-level” moderator).

- Also unmentioned are potential socioeconomic factors contributing to the targeted associations, despite that Co-I Lewis has published on the role of SES on alcohol consumption among lesbian women. Although it is mentioned that SES will be explored as an “identity” in future work, there is a missed opportunity to at least collect this data in the proposed study and examine relationships on an exploratory level.
- It is unclear why 15 months are required for IRB approval, material and procedure preparation, and pilot testing (which appears to already be completed). There are no details regarding estimated rates (per month or quarter) for enrollment and data collection throughout the funding period.

5. Environment:

Strengths

- The Principal Investigator and Co-Is have sufficient lab/office space and computing software/hardware at Old Dominion University to conduct the proposed work.
- Research team cohesion is promoted by close workspace proximity.

Weaknesses

- None noted.

Study Timeline:

Strengths

- None noted.

Weaknesses

- It is unclear why 15 months are required for IRB approval, material and procedure preparation, and pilot testing (which appears to already be completed). There are no details regarding estimated rates (per month or quarter) for enrollment and data collection throughout the funding period.

Protections for Human Subjects:

Acceptable Risks and/or Adequate Protections

- Risks are minimal and protections against risk adequately addressed.

Data and Safety Monitoring Plan (Applicable for Clinical Trials Only):

Not Applicable (No Clinical Trials)

Inclusion of Women, Minorities and Children:

- Sex/Gender: Distribution justified scientifically
- Race/Ethnicity: Distribution justified scientifically
- For NIH-Defined Phase III trials, Plans for valid design and analysis: Not applicable
- Inclusion/Exclusion of Children under 18: Excluding ages <18; justified scientifically

- 300 women aged 18-30 will be recruited, of whom 100 will be Black. This population and sample size appears in line with the research goals and is adequately justified.

Vertebrate Animals:

Not Applicable (No Vertebrate Animals)

Biohazards:

Not Applicable (No Biohazards)

Resubmission:

- The Principal Investigators have been largely responsive to the prior critiques, focusing on binge eating behaviors rather than "disordered eating", refining eligibility criteria and EMA survey schedules, providing psychometrics on the Social Processes and Sexual Minority Stress measure, increasing the sample size of Black women in each group to explore intersections between sexual minority and racial minority factors, and including alcohol consumption as a potential mediating variable.
- Some comments remain insufficiently addressed (inclusion of other demographic factors, similarities in associations between negative affect and disordered eating in heterosexual and lesbian women, justification for the age range, condensing the timeline).

Budget and Period of Support:

Budget Modifications Recommended (in amount/time)

Recommended budget modifications or possible overlap identified:

- It is unclear why 15 months are required for study start-up/piloting/IRB; the period of support (4 years of funding) is inadequately justified as it appears that the work could be completed in 3 years.

CRITIQUE 3

Significance: 1
Investigator(s): 1
Innovation: 2
Approach: 2
Environment: 1

Overall Impact: This is a highly innovative application which seeks to address the significant public health problem of racial and sexual minority health disparities, mainly binge eating and obesity. The investigators responded thoroughly to reviewer concerns raised during their initial submission and the strength of the application has increased as a result of their responsiveness. For example, interest in exploring disparities among individuals that live at the intersection of multiple minority identities is an emerging scientific priority and this application responded beautifully to the previous reviewer’s suggestion that they add an aim focused on exploring individuals with both racial and sexual minority identities; this aim is both significant and innovative. Additional marked strengths of this application include the strong team of investigators and the environment in which the work will be done. Further, the significant amount of pilot testing that has been conducted prior to this application submission helps

to ensure that the investigative team will be successful in carrying out the work as proposed. All noted weaknesses are minor.

1. Significance:

Strengths

- This work importantly addresses significant health problems (obesity, binge eating) among particularly vulnerable populations (lesbian women, black women).
- An important strength is the choice to examine the intersection of racial and sexual minority identities is a huge strength.
- Proposal aligns with stated public health priorities (IOM reports, NIH Strategic Plan) to reduce sexual minority health disparities.
- This work offers high potential to inform existing binge eating and obesity treatment programs that currently fail to tailor treatment to lesbian women, as well as women living with both racial and sexual minority identities.

Weaknesses

- None noted.

2. Investigator(s):

Strengths

- The study Principal Investigator has a strong research trajectory, including an expertise in EMA research as well as experience conducting research on binge eating and sexual minority health, positioning her well to lead this application as it is written.
- The study team has a strong history of successful collaborations on related projects; the diversity in expertise married with common research goals will serve this project well.
- Inclusion of Dr. Braitman on the study team is a strength given her expertise in multilevel modeling which will be needed to lead analysis of this proposed work.

Weaknesses

- None noted.

3. Innovation:

Strengths

- This application identifies significant gaps in our understanding of binge eating within the context of daily life for Lesbian women and seeks to fill these gaps with the use of innovative EMA methods to explore multiple “within person” questions about the predictors and correlates of binge eating in daily life. While the use of EMA is not always innovative (as mentioned by a previous reviewer) this application leverages the unique characteristics of EMA methods to answer questions that would otherwise go unanswered.
- Examination of binge eating behaviors among women at intersection of minority race and sexual identity is highly innovative – first study to examine binge eating within the context of daily life among this population.
- Focus on theory-based sexual minority-specific factors at multiple levels with the opportunity to explore between person AND within person variance in predictors of binge eating behavior.

Weaknesses

- Timed biomarker assessment of stress (cortisol via saliva swab) paired with EMA measures could add dramatically to study innovation.

4. Approach:

Strengths

- Approach is guided by minority stress theories, as well as informed by significant pilot work conducted by the team of investigators. Successful pilot testing on nearly all aspects of the proposed study.
- Use of EMA methods to allow for examination of within-person associations (in addition to between person associations) will shed light on individual level processes related to binge eating in daily life.
- Use of multiple marketing groups to assist with recruitment of participants; demonstrated previous success in recruiting women of minority sexual identify through these marketing firms in the past.
- Oversampling of Black participants to allow for examination of minority race as a potential moderator is a marked strength.

Weaknesses

- The study plans to oversample black participants (n=50 lesbian, n=50 heterosexual), but the remaining participants represent a range of racial backgrounds; it is unclear how participants from non-black minority racial groups (e.g. Native American) will be handled within data analysis focused on the role of minority race as a moderator.

5. Environment:

Strengths

- The academic environment (Old Dominian) is well suited to carry out the proposed work.
- Decision to work with multiple marketing groups to facilitate recruitment is a strength, particularly given the need to recruit individuals with multiple minority identities.

Weaknesses

- None noted.

Study Timeline:

Strengths

- The proposed timeline is appropriate and very well researched; allows ample time for preparation, recruitment, data collection, analysis and dissemination of findings.

Weaknesses

- Timeline includes multiple months for pilot testing measures and procedures; given the significant amount of pilot work conducted by this study team it seems that this amount of additional time might not be needed.

Protections for Human Subjects:

Acceptable Risks and/or Adequate Protections

Data and Safety Monitoring Plan (Applicable for Clinical Trials Only):

Not Applicable (No Clinical Trials)

***Inclusion of Women*, Minorities and Children:**

- Sex/Gender: Distribution justified scientifically
- Race/Ethnicity: Distribution justified scientifically
- For NIH-Defined Phase III trials, Plans for valid design and analysis: Not applicable
- Inclusion/Exclusion of Children under 18: Excluding ages <18; justified scientifically
- The proposed study will include women and minorities and exclude men; the inclusion (women and minorities) and exclusion (men) of these individuals is intentional and the proposed distribution is justified scientifically. The proposed study will exclude individuals under 18; this proposed exclusion is justified scientifically.

Vertebrate Animals:

Not Applicable (No Vertebrate Animals)

Biohazards:

Not Applicable (No Biohazards)

Resubmission:

- The investigators responded thoroughly to all previously mentioned weaknesses and thus has improved upon their original application significantly.

Budget and Period of Support:

Recommended budget modifications or possible overlap identified:

- The pilot work accomplished to date would seem to indicate that the time allocated for further pilot testing is excessive

THE FOLLOWING SECTIONS WERE PREPARED BY THE SCIENTIFIC REVIEW OFFICER TO SUMMARIZE THE OUTCOME OF DISCUSSIONS OF THE REVIEW COMMITTEE, OR REVIEWERS' WRITTEN CRITIQUES, ON THE FOLLOWING ISSUES:

PROTECTION OF HUMAN SUBJECTS: ACCEPTABLE

INCLUSION OF WOMEN PLAN: ACCEPTABLE

INCLUSION OF MINORITIES PLAN: ACCEPTABLE

INCLUSION OF CHILDREN PLAN: ACCEPTABLE

COMMITTEE BUDGET RECOMMENDATIONS: The committee recommends a shortened time line or budget review of time and salary expended for pilot testing in list of the extensive pilot testing accomplished to date; further pilot testing deemed excessive.

Footnotes for 1 R01 MD012598-01A1; PI Name: HERON, KRISTIN E

NIH has modified its policy regarding the receipt of resubmissions (amended applications). See Guide Notice NOT-OD-14-074 at http://grants.nih.gov/grants/guide/notice-files/NOT-OD-14-074.html. The impact/priority score is calculated after discussion of an application by averaging the overall scores (1-9) given by all voting reviewers on the committee and multiplying by 10. The criterion scores are submitted prior to the meeting by the individual reviewers assigned to an application, and are not discussed specifically at the review meeting or calculated into the overall impact score. Some applications also receive a percentile ranking. For details on the review process, see http://grants.nih.gov/grants/peer_review_process.htm#scoring.

MEETING ROSTER

Psychosocial Risk and Disease Prevention Study Section
Risk, Prevention and Health Behavior Integrated Review Group
CENTER FOR SCIENTIFIC REVIEW
PRDP
06/11/2018 - 06/12/2018

Notice of NIH Policy to All Applicants: Meeting rosters are provided for information purposes only. Applicant investigators and institutional officials must not communicate directly with study section members about an application before or after the review. Failure to observe this policy will create a serious breach of integrity in the peer review process, and may lead to actions outlined in NOT-OD-14-073 at https://grants.nih.gov/grants/guide/notice-files/NOT-OD-14-073.html and NOT-OD-15-106 at https://grants.nih.gov/grants/guide/notice-files/NOT-OD-15-106.html, including removal of the application from immediate review.

CHAIRPERSON(S)

SPRING, BONNIE, PHD
PROFESSOR
DEPARTMENT OF PREVENTIVE MEDICINE
NORTHWESTERN UNIVERSITY
CHICAGO, IL 60611

MEMBERS

APPELHANS, BRADLEY M, PHD
ASSOCIATE PROFESSOR
DEPARTMENT OF PREVENTIVE MEDICINE
RUSH UNIVERSITY MEDICAL CENTER
CHICAGO, IL 60612

BARKIN, SHARI, MD *
PROFESSOR OF PEDIATRICS
DIVISION OF GENERAL PEDIATRICS
DEPARTMENT OF PEDIATRICS
VANDERBILT UNIVERSITY
NASHVILLE, TN 37232

BEFORT, CHRISTIE, PHD
ASSOCIATE PROFESSOR
DEPARTMENT OF PREVENTIVE MEDICINE
AND PUBLIC HEALTH
UNIVERSITY OF KANSAS MEDICAL CENTER
KANSAS CITY, KS 66160

BOUTELLE, KERRI N, PHD *
PROFESSOR
PEDIATRICS, FAMILY MEDICINE AND PUBLIC HEALTH
UNIVERSITY OF CALIFORNIA, SAN DIEGO
LA JOLLA, CA 92037

BUMAN, MATTHEW P, PHD
ASSOCIATE PROFESSOR
COLLEGE OF HEALTH SOLUTIONS
ARIZONA STATE UNIVERSITY
PHOENIX, AZ 85004

CAAN, BETTE J, DRPH *
SENIOR RESEARCH SCIENTIST
DIVISION OF RESEARCH
KAISER PERMANETE
OAKLAND, CA 94612

DEMARK-WAHNEFRIED, WENDY, PHD
PROFESSOR AND WEBB ENDOWED CHAIR
DEPARTMENT OF NUTRITION SCIENCES
UNIVERSITY OF ALABAMA AT BIRMINGHAM
BIRMINGHAM , AL 35294

DUTTON, GARETH R, PHD
ASSOCIATE PROFESSOR
DEPARTMENT OF MEDICINE
DIVISION OF PREVENTIVE MEDICINE
UNIVERSITY OF ALABAMA AT BIRMINGHAM
BIRMINGHAM, AL 35205

FISCELLA, KEVIN, MD, MPH
PROFESSOR
DEPARTMENT OF FAMILY MEDICINE
SCHOOL OF MEDICINE
UNIVERSITY OF ROCHESTER
ROCHESTER, NY 14620

FITZPATRICK, STEPHANIE LENAY, PHD *
INVESTIGATOR
CENTER FOR HEALTH RESEARCH
KAISER PERMANENTE
PORTLAND, OR 97227

FOCHT, BRIAN CARL, PHD *
PROFESSOR
COLLEGE OF EDUCATION
AND HUMAN ECOLOGY
THE OHIO STATE UNIVERSITY
COLUMBUS, OH 43210

FRANCIS, LORI ANNE, PHD
ASSOCIATE PROFESSOR
DEPARTMENT OF BIOBEHAVIORAL HEALTH
PENNSYLVANIA STATE UNIVERSITY
UNIVERSITY PARK, PA 16802

FUEMMELER, BERNARD F, PHD
PROFESSOR AND GORDON D. GINDER, MD CHAIR IN CANCER RESEARCH
DEPARTMENT OF HEALTH BEHAVIOR AND POLICY
MASSEY CANCER CENTER
VIRGINIA COMMONWEALTH UNIVERSITY
RICHMOND, VA 23298

HOLSEN, LAURA MCGRATH, PHD *
ASSISTANT PROFESSOR
DEPARTMENT OF PSYCHIATRY
HARVARD MEDICAL SCHOOL
HARVARD UNIVERSITY
BOSTON, MA 02115

JAKICIC, JOHN M, PHD
PROFESSOR AND CHAIR
DEPARTMENT OF HEALTH
AND PHYSICAL ACTIVITY
UNIVERSITY OF PITTSBURGH
PITTSBURGH, PA 15261

LEWIS, BETH A, PHD *
PROFESSOR & DIRECTOR
SCHOOL OF KINESIOLOGY
UNIVERSITY OF MINNESOTA
MINNEAPOLIS, MN 55455

LOTH, KATIE ANN, PHD *
ASSISTANT PROFESSOR
DEPARTMENT OF FAMILY MEDICINE
AND COMMUNITY HEALTH
UNIVERSITY OF MINNESOTA
MINNEAPOLIS , MN 55414

MUCCI, LORELEI, SCD *
ASSOCIATE PROFESSOR
DEPARTMENT OF EPIDEMIOLOGY
HARVARD SCHOOL OF PUBLIC HEALTH
HARVARD UNIVERSITY
BOSTON, MA 02115

NAPOLITANO, MELISSA A, PHD
PROFESSOR
DEPARTMENTS OF PREVENTION
AND COMMUNITY HEALTH, EXERCISE
AND NUTRITION SCIENCES
GEORGE WASHINGTON UNIVERSITY
WASHINGTON, DC 20052

NEELON, SARA ELIZABETH, PHD
ASSOCIATE PROFESSOR
DEPARTMENT OF HEALTH
BEHAVIOR AND SOCIETY
JOHNS HOPKINS SCHOOL OF PUBLIC HEALTH
BALTIMORE , MD 21205

NIX, ROBERT, PHD *
ASSOCIATE PROFESSOR
HUMAN DEVELOPMENT AND FAMILY STUDIES
SCHOOL OF HUMAN ECOLOGY
UNIVERSITY OF WISCONSIN-MADISON
MADISON, WI 53706

NOCK, NORA L, PHD
ASSOCIATE PROFESSOR
DEPARTMENT OF EPIDEMIOLOGY
AND BIOSTATISTICS
CASE WESTERN RESERVE UNIVERSITY
CLEVELAND , OH 44106

PAUL, IAN M, MD
PROFESSOR
DEPARTMENT OF PEDIATRICS
COLLEGE OF MEDICINE
PENNSYLVANIA STATE UNIVERSITY
HERSHEY, PA 17033

RAYNOR, HOLLIE A, PHD
PROFESSOR
DEPARTMENT OF NUTRITION
UNIVERSITY OF TENNESSEE
KNOXVILLE, TN 37996

ROSAS, LISA GOLDMAN, PHD *
ASSISTANT PROFESSOR
DIVISIONS OF EPIDEMIOLOGY AND PRIMARY CARE
AND POPULATION HEALTH
RESEARCH INSTITUTE
STANFORD UNIVERSITY SCHOOL OF MEDICINE
PALO ALTO, CA 94305

SAELENS, BRIAN E, PHD *
PROFESSOR
CENTER FOR CHILD HEALTH, BEHAVIOR
AND DEVELOPMENT
SEATTLE CHILDREN'S HOSPITAL RESEARCH INSTITUTE
UNIVERSITY OF WASHINGTON
SEATTLE, WA 98101

SCHNEIDER, MARGARET L, PHD
PROFESSOR
DEPARTMENTS OF URBAN PLANNING AND PUBLIC POLICY
INSTITUTE FOR CLINICAL AND TRANSLATIONAL SCIENCE
UNIVERSITY OF CALIFORNIA, IRVINE
IRVINE, CA 92617

SEVICK, MARY A, SCD
PROFESSOR
DEPARTMENT OF POPULATION HEALTH
SCHOOL OF MEDICINE
NEW YORK UNIVERSITY
NEW YORK, NY 10016

WEISSMAN, RUTH STRIEGEL, PHD *
PROFESSOR
DEPARTMENT OF PSYCHOLOGY
WESLEYAN UNIVERSITY
MIDDLETOWN, CT 06459

WILLIAMS, DAVID M, PHD
ASSOCIATE PROFESSOR
DEPARTMENT OF BEHAVIORAL AND SOCIAL SCIENCES
CENTER FOR HEALTH EQUITY RESEARCH
BROWN UNIVERSITY SCHOOL OF PUBLIC HEALTH
PROVIDENCE, RI 02912

SCIENTIFIC REVIEW OFFICER

FITZSIMMONS, STACEY, PHD, MPH
SCIENTIFIC REVIEW OFFICER
CENTER FOR SCIENTIFIC REVIEW
NATIONAL INSTITUTES OF HEALTH
BETHESDA, MD 20892

EXTRAMURAL SUPPORT ASSISTANT

WALKER, SHEENA
EXTRAMURAL SUPPORT ASSISTANT (INTERN)
CENTER FOR SCIENTIFIC REVIEW
NATIONAL INSTITTUES OF HEALTH
BETHESDA, MD 20892

* Temporary Member. For grant applications, temporary members may participate in the entire meeting or may review only selected applications as needed.

Consultants are required to absent themselves from the room during the review of any application if their presence would constitute or appear to constitute a conflict of interest.
